# Real-space refinement in *PHENIX* for cryo-EM and crystallography

**DOI:** 10.1107/S2059798318006551

**Published:** 2018-05-30

**Authors:** Pavel V. Afonine, Billy K. Poon, Randy J. Read, Oleg V. Sobolev, Thomas C. Terwilliger, Alexandre Urzhumtsev, Paul D. Adams

**Affiliations:** a Molecular Biophysics and Integrated Bioimaging Division, Lawrence Berkeley National Laboratory, Berkeley, CA 94720, USA; b Department of Physics and International Centre for Quantum and Molecular Structures, Shanghai University, Shanghai 200444, People’s Republic of China; c Cambridge Institute for Medical Research, University of Cambridge, Wellcome Trust/MRC Building, Hills Road, Cambridge CB2 0XY, England; d Bioscience Division, Los Alamos National Laboratory, Los Alamos, NM 87545, USA; e New Mexico Consortium, Los Alamos, NM 87545, USA; f Faculté des Sciences et Technologies, Université de Lorraine, BP 239, 54506 Vandoeuvre-les-Nancy, France; g Centre for Integrative Biology, IGBMC, CNRS–INSERM–UdS, 1 Rue Laurent Fries, BP 10142, 67404 Illkirch, France; h Department of Bioengineering, University of California Berkeley, Berkeley, California, USA

**Keywords:** real-space refinement, cryo-EM, crystallography, map interpolation, atomic-centered targets, *PHENIX*

## Abstract

This article describes the implementation of real-space refinement in the *phenix.real_space_refine* program from the *PHENIX* suite. The use of a simplified refinement target function enables very fast calculation, which in turn makes it possible to identify optimal data-restraint weights as part of routine refinements with little runtime cost. Refinement of atomic models against low-resolution data benefits from the inclusion of as much additional information as is available. In addition to standard restraints on covalent geometry, *phenix.real_space_refine* makes use of extra information such as secondary-structure and rotamer-specific restraints, as well as restraints or constraints on internal molecular symmetry. The re-refinement of 385 cryo-EM-derived models available in the Protein Data Bank at resolutions of 6 Å or better shows significant improvement of the models and of the fit of these models to the target maps.

## Abstract 2

A description is provided of the implementation of real-space refinement in the *phenix.real_space_refine* program from the *PHENIX* suite and its application to the re-refinement of cryo-EM-derived models.

## 1. Introduction   

**Figure 1:**
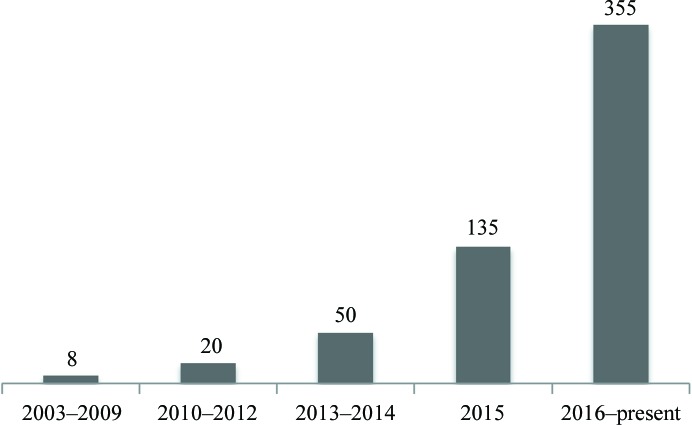
Number of cryo-EM-derived models in the PDB at resolutions of 6 Å or better.

Improvements in the cryo-electron microscopy (cryo-EM) technique have led to a rapid increase in the number of high-resolution three-dimensional reconstructions that can be interpreted with atomic models (Fig. 1 ▸). This has prompted a number of new developments in *PHENIX* (Adams *et al.*, 2010 ▸) to support the method, from model building (Terwilliger, Adams *et al.*, 2018 ▸), map improvement (Terwilliger, Sobolev *et al.*, 2018 ▸) and refinement (Afonine *et al.*, 2013 ▸) to model validation (Afonine *et al.*, 2018 ▸). In this manuscript, we focus on atomic model refinement using a map (primarily cryo-EM, but the same algorithms and software are also applicable to crystallographic maps).

Model refinement is an optimization problem and as such it requires the definition of three entities (for reviews, see Tronrud, 2004 ▸; Watkin, 2008 ▸; Afonine *et al.*, 2012 ▸, 2015 ▸). Firstly, the model, *i.e.* a mathematical construct that explains the experimental data, with an associated set of refinable parameters: in this case an atomic model with coordinates whose positions can be varied to improve the fit to the data. Seondly, the target function that links the model parameters to the experimental data: this function scores model-to-data fit and therefore guides refinement. Finally, an optimization method that changes the values of refinable model parameters such that the model agreement with the experimental data is improved. In *PHENIX*, gradient methods are used through L-BFGS (Liu & Nocedal, 1989 ▸) for this goal. If the target function is expressed through diffraction intensities or structure factors, refinement is usually referred to as reciprocal-space, or Fourier-space, refinement (FSR). Alternatively, a target function may be formulated in terms of a map: a Fourier synthesis in the case of crystallography or a three-dimensional reconstruction from projections in the case of cryo-EM. Such refinement is referred to as real-space refinement (RSR). In both cases the targets are the sums over a large number of similar terms corresponding to either reflections (FSR) or map grid points (RSR). A key methodological difference is that for RSR each term depends on only a few atoms, while for FSR each term depends on all model parameters. Most modern macromolecular refinement programs were developed for crystallographic data and therefore perform refinement in reciprocal space, at least as their main mode of operation (see Table 1 in Afonine *et al.*, 2015 ▸). This work focuses on the real-space refinement of coordinates of atomic models.

In cryo-EM studies real-space refinement is a natural choice because a three-dimensional map is the output of the single-particle image-reconstruction method (see, for example, Frank, 2006 ▸) and does not change in a fundamental way as the atomic model is improved. This is not the case for crystallo­graphy, where the experimental data are diffraction intensities, and the associated and vital phase information has to be obtained indirectly. In crystallography, obtaining the best phases typically involves their calculation from atomic models, in turn making the resulting maps model-biased (see, for example, Hodel *et al.*, 1992 ▸). Although FSR methods are predominant in crystallographic refinement, RSR is attractive in some contexts as it makes it possible to refine parts of the model locally and fast, and model incompleteness does not influence refinement as it does for FSR (Lunin *et al.*, 2002 ▸). For this reason RSR has been particularly popular in the context of interactive model-building software such as *FRODO*, *O* (Jones, 1978 ▸; Jones *et al.*, 1991 ▸), *MAIN* (Turk, 2013 ▸) and *Coot* (Emsley & Cowtan, 2004 ▸; Emsley *et al.*, 2010 ▸).

In the case of cryo-EM an atomic model can also be refined using a reciprocal-space target. This can be achieved by converting the map into Fourier coefficients. These Fourier coefficients can then be used in reciprocal-space refinement using standard refinement protocols that are well established for crystallographic structure refinement (see, for example, Cheng *et al.*, 2011 ▸; Baker *et al.*, 2013 ▸; Brown *et al.*, 2015 ▸). We note, however, that unless the map is converted to the full corresponding set of Fourier coefficients (and not a subset containing only a sphere limited to the stated resolution) this conversion may not be lossless.

To address the emerging structure-refinement needs of the rapidly growing field of cryo-EM, the *phenix.real_space_refine* program (Afonine *et al.*, 2013 ▸), which is capable of the refinement of atomic models against maps, has been introduced into the *PHENIX* suite. It is not limited to cryo-EM and can also be used in crystallographic refinement (X-ray, electron or neutron). In this paper, we describe the implementation of the *phenix.real_space_refine* program and demonstrate its performance by applications to simulated data and to cryo-EM models in the PDB (Bernstein *et al.*, 1977 ▸; Berman *et al.*, 2000 ▸) and corresponding maps in the EMDB (Henrick *et al.*, 2003 ▸). This is a work in progress, and further details and advances will be reported as the program evolves. To date, *phenix.real_space_refine* has been used in a number of documented structural studies (see, for example, Fischer *et al.*, 2015 ▸; Shalev-Benami *et al.*, 2016 ▸; Chua *et al.*, 2016 ▸; Ahmed *et al.*, 2016 ▸; Yang *et al.*, 2016 ▸; Gao *et al.*, 2016 ▸; Chen *et al.*, 2016 ▸; Bhardwaj *et al.*, 2016 ▸; Lokareddy *et al.*, 2017 ▸; Hryc *et al.*, 2017 ▸; Ahmed *et al.*, 2017 ▸; Demo *et al.*, 2017 ▸; Paulino *et al.*, 2017 ▸; Liu *et al.*, 2017 ▸).

## 2. Methods   

### 2.1. Refinement flowchart   

**Figure 2:**
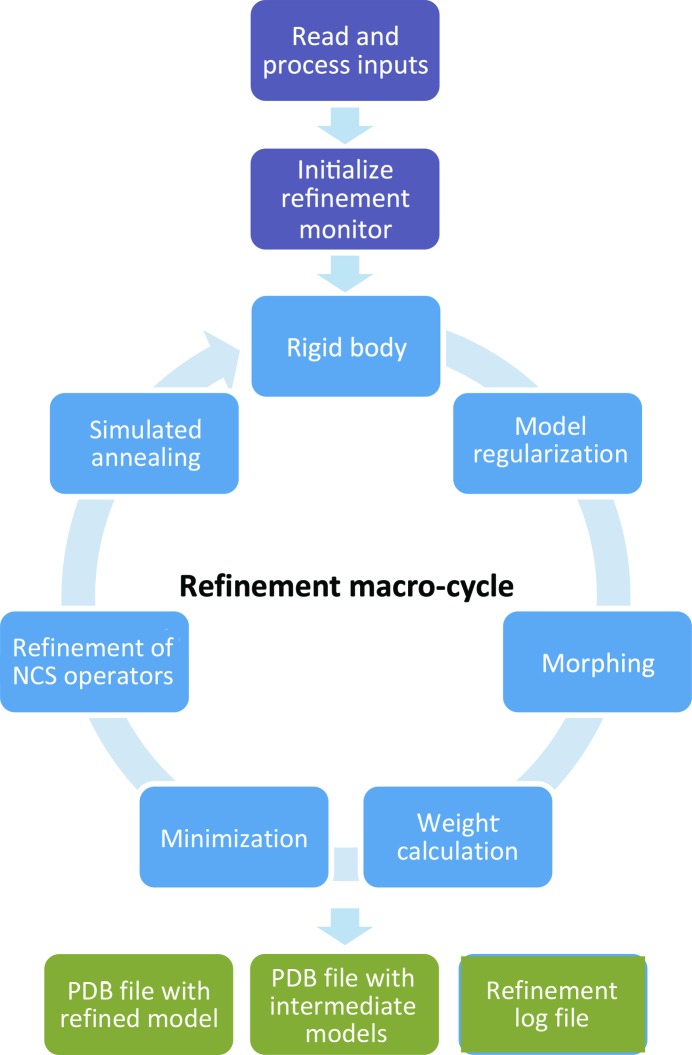
Flowchart for *phenix.real_space_refine*.

Fig. 2 ▸ shows the model-refinement flowchart as it is implemented in *phenix.real_space_refine*. This is very similar to the reciprocal-space refinement workflow implemented in *phenix.refine* (see Fig. 1 in Afonine *et al.*, 2012 ▸).

The program begins by reading a model file, in PDB or mmCIF format, map data (as an actual map in MRC/CCP4 format or as Fourier map coefficients in MTZ format) and other parameters, such as resolution (if a map is provided) or additional restraint definitions for novel ligands, internal molecular symmetry (*e.g.* NCS in crystallography) or secondary structure. Once inputs have been read, the program proceeds to calculations that constitute a set of tasks repeated multiple times (macro-cycles). Tasks to be performed during the refinement are defined by the program automatically and/or by the user. In its default mode the program will only perform gradient-driven minimization of the entire model. Other nondefault tasks allow optimization using simulated annealing (SA; Brünger *et al.*, 1987 ▸), morphing (Terwilliger *et al.*, 2013 ▸), rigid-body refinement (see Afonine *et al.*, 2009 ▸ and references therein) and systematic residue side-chain optim­izations using grid searches in torsion χ-angle space (Oldfield, 2001 ▸). Parts of the model related by internal symmetry are determined automatically, if available, or can be defined by the user. In the presence of such internal symmetry, restraints or constraints can be applied between the coordinates of related molecules. The operators relating molecules can also be refined. The result of refinement, *i.e.* the refined model, is output as a file in PDB or mmCIF format.

Central to almost all tasks performed within a refinement macro-cycle is the target function. Its choice is the key for the success of refinement, *i.e.* efficient convergence to an improved model. Also of the same importance is the assessment of refinement progress by quantifying model quality and the goodness of model-to-map fit throughout the entire process. Some relevant points are discussed below.

### 2.2. Refinement target function   

Macromolecular cryo-EM or crystallographic experimental data are almost always of insufficient quality to refine parameters of atomic models individually. To make refinement practical, restraints or constraints are almost always used in order to incorporate extra information into refinement, and the corresponding procedures are called restrained or constrained refinement. In restrained refinement the target function is a sum of data-based and restraints-based components:
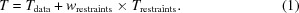
The first term scores the model-to-data fit and the second term incorporates *a priori* information about the model. The weight *w*_restraints_ balances the contribution of restraints to maximize the model-to-data fit while also obeying the *a priori* information, and an optimal choice of its value is crucial. Constrained refinement does not change the target function but rather changes (reduces) the set of independent parameters that can vary. Examples include rigid-body refinement, the use of a riding model (Sheldrick & Schneider, 1997 ▸) to parameterize the positions of H atoms in refinement or the implementation of RSR by Diamond (1971 ▸) using torsion angles as variables.

#### 2.2.1. Model-to-map target (*T*_data_)   

In RSR, the *T*_data_ term scores the fit of the model being refined to a target map. In cryo-EM the map is a three-dimensional reconstruction, while in crystallography it may be, for example, a 2*mF*_obs_ − *DF*_model_ map (Read, 1986 ▸).

It is possible to express the difference between the two maps in the integral form (see, for example, Diamond, 1971 ▸)^1^

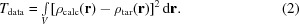
For (2) we suppose that the original target map is optimally scaled to the model map (Diamond, 1971 ▸; Chapman, 1995 ▸). In the following, we will consider the target to be essentially unchanged by manipulations that shift its value by a constant or a scale factor, as such manipulations do not change the position of the minimum of the target. If the Euclidean norms of ρ_tar_(**r**) and ρ_calc_(**r**) are conserved during refinement [*i.e.* if 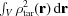 = constant, as will be the case when the target map itself does not change, and if 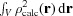 = constant, which will be true if the overlap of atomic densities does not change] then minimization of (2) is equivalent to minimization of the anticorrelation target, which does not need the maps to be optimally scaled,
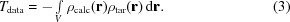
Assuming the target ρ_tar_ and model-calculated ρ_calc_ maps are provided on the same grid, a continuous integration in (2) and (3) can be replaced with a numeric integration over the regular grid on which the maps are available (see, for example, Diamond, 1971 ▸),
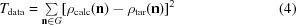
or
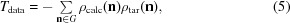
respectively. The set *G* of grid nodes used to calculate the targets (*i.e.* the integration volume) is either the whole map or an envelope (mask) surrounding the whole atomic model or its part that is subject to refinement.

To match the finite resolution of the target map in (5) accurately, several steps are required to compute the model map. Firstly, the model map distribution is calculated using one of the available approximations (Sears, 1992 ▸; Maslen *et al.*, 1992 ▸; Waasmaier & Kirfel, 1995 ▸; Grosse-Kunstleve *et al.*, 2004 ▸; Peng *et al.*, 1996 ▸; Peng, 1998 ▸). A set of Fourier coefficients is then calculated from the distribution up to the resolution limit specified by the target map.^2^ Finally, a subset of these coefficients is used to calculate the model Fourier synthesis ρ_calc_ that can then be used in (5). This synthesis is a representation of a model image at a given resolution. A typical refinement may require hundreds or even thousands of such model-image calculations, which are computationally expensive, involving two Fourier transforms.

Alternatively, a model map may be calculated from the atomic model directly as a sum of individual contributions of *M* atoms, with each contribution being a Fourier image (or its approximation) of the corresponding atom at a given resolution (see, for example, Diamond, 1971 ▸; Lunin & Urzhumtsev, 1984 ▸; Chapman, 1995 ▸; Mooij *et al.*, 2006 ▸; Sorzano *et al.*, 2015 ▸). While this is much faster than the previous method, it may be less accurate and still be computationally expensive, especially for large models.

A numeric integration over the whole map (5) can be simplified by the integration exploring the volume directly around the atomic centers **r**_*m*_, *m* = 1, … *M*:
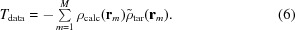
Here, 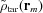 are the values interpolated from the nearby grid node values ρ_tar_(**n**) to the atomic centers **r**_*m*_ (Appendices *A* [App appa] and *B* [App appb]). Neglecting the local variation of the model map at the atomic centers (*e.g.* at low resolution) and thus supposing ρ_calc_(**r**_*m*_) ≃ constant for all *m*, the target simplifies further as (Rossmann, 2000 ▸; Rossmann *et al.*, 2001 ▸)
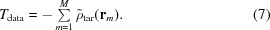
The hypothesis ρ_calc_(**r**_*m*_) ≃ constant seems to be reasonable at low resolution, when a calculated map can be considered to be rather flat. On the other hand, minimization of (7) is essentially a fitting of atoms to the nearest peaks of the target map, which seems to be appropriate at high resolution as well. We show below (§[Sec sec3]3) that indeed this target function is efficient over a large resolution range; Appendix *B* [App appb] supports this observation through the equivalence of targets (7) and (5) when taking map blurring/sharpening into account. If the difference in atomic size cannot be neglected, this target function can be modified to
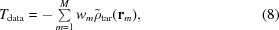
where *w_m_* is an atom-specific weight. For example, *w_m_* can be the electron number of the corresponding atom or it can be set negative for O atoms of Asp and Glu residues in the case of cryo-EM or for atoms that have a negative scattering length (such as hydrogen) in the case of neutron diffraction data. Clearly, for most of the macromolecular structures under consideration here these atom-centered targets are nearly the same, and for simplicity in the following we refer only to (7) unless otherwise stated. The computational cost of (7) is proportional, with a very small coefficient, to the number of atoms and therefore these targets are much faster to calculate compared with (5), making it advantageous for the refinement of large models. Unlike (4) or (5), the computational cost of (7) or (8) does not depend on the resolution or map-sampling rate. Essentially, target (5) optimizes the fit of the shape between model-calculated and experimental maps, while target (7) simply guides atoms to the nearest peaks in the experimental map. Therefore, refinement using (5) can produce a more accurate model-to-map fit. An optimal refinement protocol may consist of using target (7) for routine refinements and using (5) for the final refinement.

#### 2.2.2. Restraints (*T*_restraints_)   

In restrained refinement, extra information is introduced through the term *T*_restraints_ with some weight (1). This extra term restrains model parameters to be similar, but not necessarily identical, to some reference values. At high to medium resolutions of approximately 3 Å or better, a standard set of restraints as implemented in *PHENIX* includes (Grosse-Kunstleve & Adams, 2004 ▸) restraints on covalent bond lengths and angles, dihedral angles, planarity and chirality restraints, and a nonbonded repulsion term. However, at lower resolutions the amount of experimental data is insufficient to preserve the geometry characteristics of a higher level of structural organization (such as secondary structure), and therefore including extra information (restraints or constraints) to help to produce a chemically meaningful model is desirable. These extra restraints or constraints may include similarity of related copies (NCS in the case of crystallography), restraints on secondary structure and restraints to one or more external reference models (for implementation details in *PHENIX*, see Headd *et al.*, 2012 ▸, 2014 ▸; Sobolev *et al.*, 2015 ▸). *phenix.real_space_refine* can use the following extra restraints and constraints.

(i) Distance and angle restraints on hydrogen-bond patterns for protein helices and sheets and DNA/RNA base pairs.
(ii) Torsion-angle restraints on idealized protein secondary-structure fragments.
(iii) Restraints to maintain stacking bases in RNA/DNA parallel.
(iv) Ramachandran plot restraints.
(v) Amino-acid side-chain rotamer-specific restraints.
(vi) C^β^ deviation restraints.
(vii) Reference-model restraints, where a reference model may be a similar structure of better quality or the initial position of the model being refined.
(viii) Similarity restraints in torsion or Cartesian space.
(ix) NCS constraints.

#### 2.2.3. Relative weight   

The relative weight *w*_restraints_ is chosen such that the model fits the map as well as possible while maintaining reasonable deviations from ideal covalent bond lengths and angles. In *PHENIX*, *w*_restraints_ for RSR is determined by systematically trying a range of plausible values and performing a short refinement for each trial value. A similar procedure in FSR would be very computationally expensive because for each trial value of *w*_restraints_ the whole structure would need to be used. In RSR this is computationally feasible using (7) but not (5). The weight-calculation procedure implemented in *phenix.real_space_refine* splits the model into a set of randomly chosen segments, each one a few residues long. After trial refinements of each segment with different weights, the best weight is defined as the one that results in a model possessing reasonable bond and angle root-mean-square deviations (r.m.s.d.s) and that has the best model-to-map fit among all trial weights. The obtained array of best weights for all fragments is filtered for outliers and the average weight is calculated and defined as the best weight for the final refinement. This calculation typically takes less than a minute on an ordinary computer and is independent of the size of the structure or map. Instead of computing an average single weight for the entire model, this protocol can be extended (work in progress) to calculate and use different weights for different parts of the map, accounting for variations in local map quality.

### 2.3. Evaluation of refinement progress and results   

It is recognized that model validation (see, for example, Brändén & Jones, 1990 ▸; Read *et al.*, 2011 ▸; Wlodawer & Dauter, 2017 ▸) is a critical step in structure determination, and a number of corresponding tools have been developed in crystallo­graphy (see, for example, Chen *et al.*, 2010 ▸; Read *et al.*, 2011 ▸; Gore *et al.*, 2017 ▸; Williams *et al.*, 2018 ▸ and references therein) and some in cryo-EM studies (see, for example, Henderson *et al.*, 2012 ▸; Tickle, 2012 ▸; Lagerstedt *et al.*, 2013 ▸; Barad *et al.*, 2015 ▸; Pintilie *et al.*, 2016 ▸; Joseph *et al.*, 2017 ▸, Afonine *et al.*, 2018 ▸). Generally, the process consists of assessing data, model quality and model-to-data fit quality, and is performed locally and globally. At the stage of refining a model we assume that the intrinsic data quality has already been evaluated, and only model quality and model-to-data fit need to be monitored.

The methods and tools to evaluate the geometric quality of a model are the same in crystallography and in cryo-EM. For example, the *PHENIX* comprehensive validation program provides an extensive report on model quality, making extensive use of the *MolProbity* validation algorithms (Chen *et al.*, 2010 ▸; Richardson *et al.*, 2018 ▸). In crystallography, the model-to-data fit is quantified by crystallographic *R* and *R*_free_ (Brünger, 1992 ▸) factors, which are global reciprocal-space metrics. In cryo-EM, model and data validation is currently performed by the comparison of complex Fourier coefficients in resolution shells; these coefficients are calculated from the model and from the full map or half-maps; different masks can be applied prior to calculation of these coefficients. Also in real space the model-to-data fit can be evaluated locally or globally by various correlation coefficients between a model-calculated map and the experimentally derived map (Urzhumtsev *et al.*, 2014 ▸; Afonine *et al.*, 2018 ▸). Some of these tools are used in §3.2, where models extracted from the PDB are refined against experimental cryo-EM maps.

## 3. Results   

### 3.1. Test refinements with simulated data   

Below, we illustrate the performance of refinement at different resolutions and map sharpnesses and using atomic models with various amounts of error in the coordinates. All refinements were performed using refinement target (1) with geometry restraints included with optimal weights and data term (7). We begin with several numerical tests using simulated data. The advantage of such tests is that one can study individual effects in a setting where the answer is known.

#### 3.1.1. Preparing simulated data   

A model from the PDB (PDB entry 3vb1) was chosen as a test model. The following manipulations were made to this model prior to test calculations: (i) the model was placed in a sufficiently large *P*1 unit cell, (ii) alternative conformations were replaced with a single conformation and (iii) model geometry was regularized using the *phenix.geometry_minimization* tool until convergence. In the following, we refer to this model as a reference model. Several Fourier maps at different resolutions *d*_high_ (1, 2, 3, 4, 5 and 6 Å) were calculated from the reference model considering three different overall *B* factors of 0, 100 and 200 Å^2^; these maps mimic ρ_tar_ (18 maps in total). The maps were calculated on a grid with the step equal to *d*_high_/4. Additionally, we calculated the same maps on a much finer grid with a step of 0.2 Å; the same step was used for all maps independent of their resolution.

#### 3.1.2. Refinement of the exact reference model   

**Figure 3:**
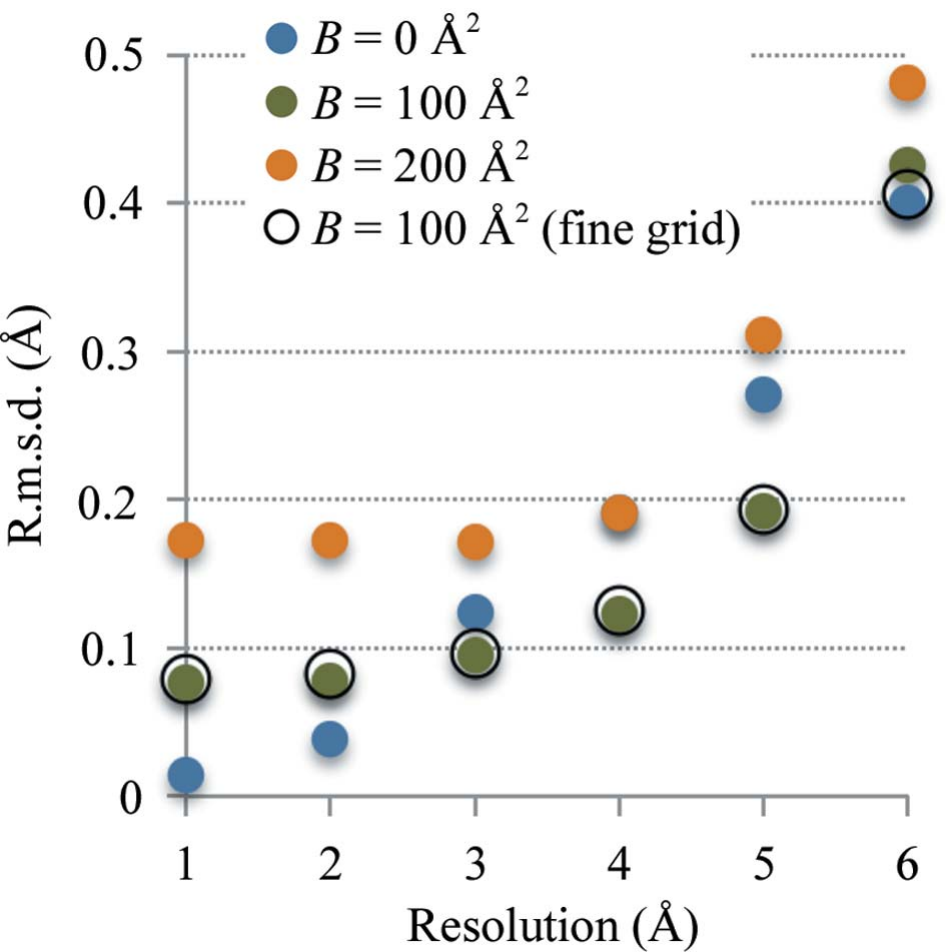
Refinement of the exact model against 18 maps computed as described in §[Sec sec3.1.1]3.1.1. Each circle shows the root-mean-square deviation between the refined model and the reference model. Blue, green and orange full circles correspond to maps with overall *B* factors of 0, 100 and 200 Å^2^, respectively. Open circles correspond to the map with an overall *B* factor of 100 Å^2^ computed on the finer grid with a step of 0.2 Å. See §[Sec sec3.1.2]3.1.2 for details.

Firstly, we refined the reference model against finite-resolution maps calculated from this model, as described in §[Sec sec3.1.1]3.1.1. While the reference model corresponds to the minimum of (5), this is not the case for (7) because map peaks in finite resolution Fourier images do not necessarily correspond to atomic centers. Therefore, it is expected that refinement using (7) may shift the model from its original, correct, position. The goal of this test is to provide an estimate of the magnitude of these shifts after refinement. For each refined model we calculated the root-mean-square deviation (r.m.s.d.) from the reference model. Fig. 3 ▸ summarizes the result of this test. We observe the following.

(i) Refinement using a finer grid does not have any significant effect compared with using a *d*_high_/4 grid step (compare the orange dots and black circles in Fig. 3 ▸).
(ii) The r.m.s.d. increases as the resolution worsens and ranges from as low as 0.01 Å at 1 Å resolution to as high as 0.48 Å at 6 Å resolution. These r.m.s.d.s are small compared with the details that can be resolved in maps at these resolutions. This justifies the use of a target (7) that is less accurate but much faster to calculate than (5).
(iii) Map sharpness has a mixed effect. At high resolution (1–2 Å) maps corresponding to the lowest *B* of 0 Å^2^ produce more accurate results. At intermediate resolutions (3–5 Å) maps corresponding to both the lowest and the largest *B* perform worse compared with those corresponding to an intermediate value (*B* = 100 Å^2^). Maps with the largest *B* of 200 Å^2^ result in overall less accurate models. These observations suggest that depending on resolution some attenuation of map sharpness may be useful.

#### 3.1.3. Refinement of perturbed reference models   

**Figure 4:**
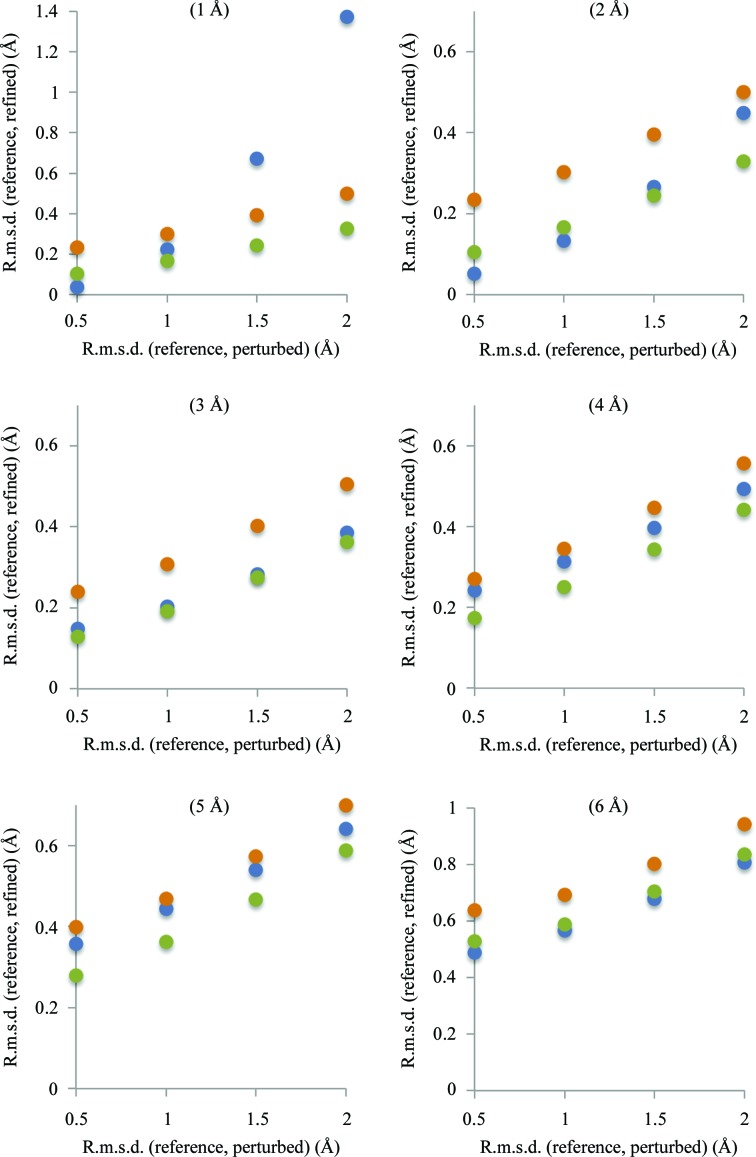
Refinement of perturbed models against maps computed as described in §[Sec sec3.1.1]3.1.1. The horizontal axis shows the r.m.s.d. between the reference model and perturbed models: 0.5, 1.0, 1.5 and 2.0 Å. The vertical axis shows the r.m.s.d. between the reference model and the refined models. Blue, green and orange full circles correspond to maps with overall *B* factors of 0, 100 and 200 Å^2^, respectively. See §[Sec sec3.1.3]3.1.3 for details.

Here, we describe tests that are similar to those in §[Sec sec3.1.2]3.1.2 except that instead of refining the reference model we refined perturbed reference models. These perturbed models were obtained by running molecular-dynamics (MD) simulations using the *phenix.dynamics* tool until a prescribed r.m.s.d. compared with the reference model was achieved. Given the stochastic nature of MD, it is possible to obtain many different models with the same r.m.s.d. from the reference model. Owing to the limited convergence radius of refinement and the finite resolution of the data, refinement of these models will not produce exactly the same refined models. Therefore, to ensure more robust statistics, for each chosen r.m.s.d. we generated an ensemble of 100 models. The r.m.s.d. values between the perturbed and reference models were chosen to be 0.5, 1.0, 1.5 and 2.0 Å. We then refined each of these 100 × 4 = 400 models against each of 18 maps (§[Sec sec3.1.1]3.1.1) calculated on a grid with a spacing of *d*_high_/4. For each refined model (from 100 × 4 × 6 × 3 = 7200 refined models) we calculated the r.m.s.d. from the reference model and then the average r.m.s.d. over the corresponding ensemble of 100 models. Fig. 4 ▸ summarizes the results of this test. We observe the following.

(i) In most cases refinement was able to significantly reduce the difference between the reference and starting perturbed models. The refinement of models with a starting r.m.s.d. of 0.5 Å gives similar results as the refinement of a nonperturbed reference model (similar r.m.s.d.).
(ii) In almost all cases using a blurred map results in less accurate refined models.
(iii) In the case of large errors (1.5–2 Å) refinement against a 1 Å resolution map corresponding to an overall *B* of 0 Å^2^ performs the worst compared with blurrier maps. This can be rationalized as the peaks on a very sharp map are narrow and sufficiently large displacements of atoms away from these peaks results in shifts that are outside the convergence radius of minimization.
(iv) At resolutions of 3–5 Å using neither very sharp nor very blurred maps produces the best results, although the effect is rather small. This suggests that there exists an optimal sharpening *B* value that is most suitable for refinement at a given resolution.

### 3.2. Refinement using data from the PDB and EMDB   

#### 3.2.1. Cryo-EM maps   

**Figure 5:**
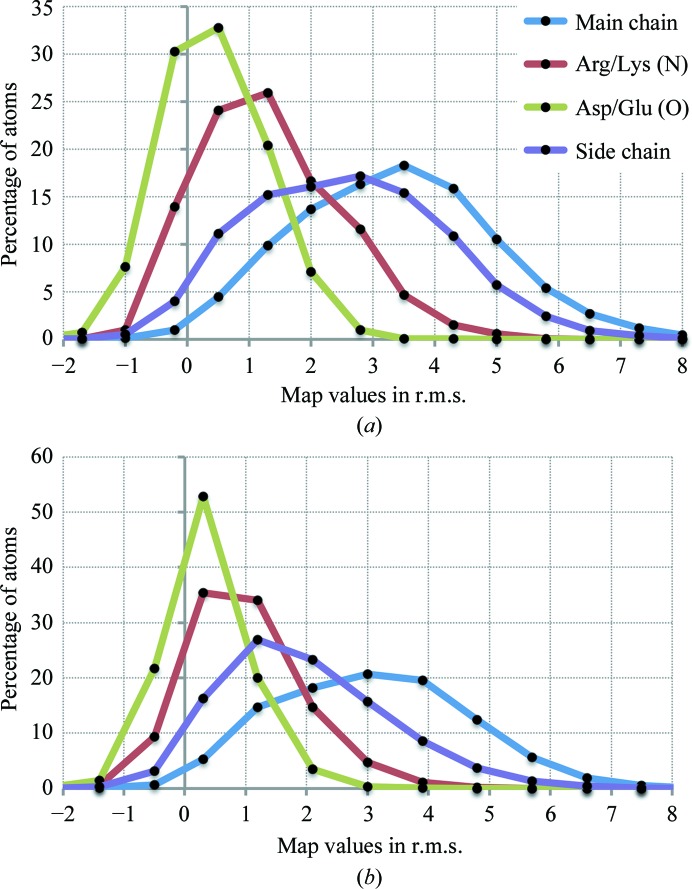
Distribution of cryo-EM map values (scaled in r.m.s.) for selected groups of atoms, considering maps at 3 Å or better (*a*) and 3–4 Å (*b*) resolution. See §[Sec sec3.2.1]3.2.1 for details.

Three-dimensional reconstructions (cryo-EM maps) represent the electric potential of the sample. Therefore, these maps are expected to have negative features around negatively charged moieties such as aspartate and glutamate (see, for example, Hryc *et al.*, 2017 ▸). Furthermore, such moieties may be susceptible to radiation damage and therefore may have a weaker footprint in the reconstructions. This may have an implication for real-space refinement that uses target (7) [or (5) if the form factors do not reproduce the negative features] because this target favors atomic shifts towards positive map peaks. To investigate this effect, we surveyed map values at atomic positions considering reconstructions at 3 Å or better and map–model correlation better than 0.8. This selected nine (map, model) pairs. Prior to calculations, we normalized all selected maps to have zero mean value and a standard deviation of 1. Fig. 5 ▸(*a*) shows the distribution of map values for four groups of atoms: main-chain atoms, side-chain O atoms of Asp and Glu residues that may be negatively charged (OD1, OD2, OE1 and OE2), side-chain atoms of Arg and Lys residues that may be positively charged (NH1, NH2 and NZ) and all other side-chain atoms. We observe that side-chain O atoms of Asp and Glu residues indeed have systematically weaker map values, with about 8% of atoms having values below a threshold of −1 times the r.m.s. of the map. Negative map values for all other kinds of atoms are greater than −0.5 r.m.s. and may be considered as noise. We note that the size and flexibility of Asp, Glu, Arg and Lys side chains are likely to contribute to systematically weaker densities for these side chains. We repeated the same analysis for maps of lower resolution (3–4 Å; Fig. 5 ▸ *b*). Here, the number of reliably observed atoms with negative features in the map is less than 1%.

This analysis shows that for the majority of cryo-EM models (resolution of 3 Å or worse) the concern about negative features in the map is rather small and is unlikely to affect the results of refinement using (7) significantly. On the other hand, the rapidly increasing number of higher resolution cryo-EM maps (better than 3 Å) is likely to highlight the limitation of (7) and to demand further improvements of the refinement target [such as using (8) with properly chosen weights].

#### 3.2.2. Default refinement   

**Figure 6:**
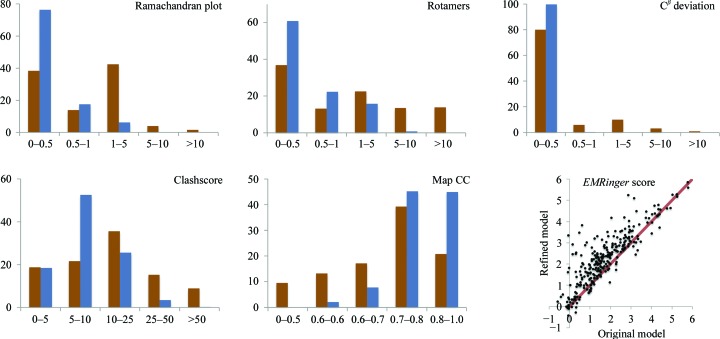
Model statistics before (brown) and after (blue) refinement using *phenix.real_space_refine*, showing Ramachandran plot and residue side-chain rotamer outliers, C^β^ deviations, *MolProbity* clashscore and model–map correlation coefficient (CC_mask_). The scatter plot shows the *EMRinger* score for the original and refined models (resolution better than 4.5 Å).

In order to test the suggested methods and demonstrate their utility, we re-refined 385 cryo-EM models from the PDB that are reported at a resolution of 6 Å or better, that have model–map correlation greater than 0.3 and that contain only residues and ligands that are known to the *PHENIX* restraint library. A number of metrics were analyzed: the model-to-map correlation coefficient CC_mask_ calculated in the map region around the model (for an exact definition, see Afonine *et al.*, 2018 ▸), the number of Ramachandran plot and rotamer outliers, excessive C^β^ deviations, the *MolProbity* clashscore (Chen *et al.*, 2010 ▸) and the *EMRinger* score (Barad *et al.*, 2015 ▸; calculated for 277 entries with maps at 4.5 Å resolution or better), all calculated for the initial models from the PDB and for the models after refinement. Default parameters were used in all refinements that, in addition to standard restraints, also include rotamer, C^β^ deviations and Ramachandran plot restraints, as well as NCS constraints where applicable (see §[Sec sec2.2.2]2.2.2). The program ran successfully, generating a refined model for all cases and highlighting the robustness of the algorithms and their implementation. In all cases we observe a substantial overall improvement of geometry metrics, such as reduced or fully eliminated Ramachandran plot and rotamer outliers, C^β^ deviations and *MolProbity* clashscore, as well as improvement of the model-to-data (map) fit (Fig. 6 ▸). Clearly, the removal of some outliers can be attributed to the use of rotamer, C^β^ deviations and Ramachandran plot restraints. Therefore, we also used an orthogonal validation metric to assess model improvement: *EMRinger* (Barad *et al.*, 2015 ▸). We observe that the overall average *EMRinger* score for the initial models is 1.73 and that for the refined models is 2.26. The improvement of the *EMRinger* score for the refined models indicates that the amino-acid side chains are more chemically realistic and better fit the map. Detailed validation or analysis of individual refinement results is outside the scope of this work, but will be important in the future to assess the impact of stereochemical restraints on models, particularly when the starting models are of very poor quality.

#### 3.2.3. Refinement against sharpened maps   

**Figure 7:**
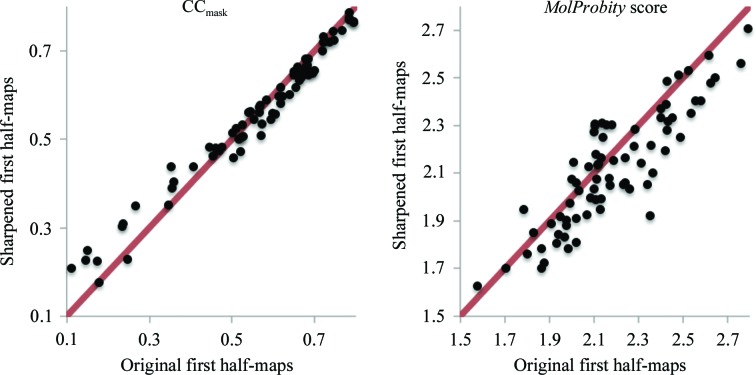
Left, correlation coefficient CC_mask_ calculated using the original second half-maps and maps calculated from models refined against the first half-maps: original (*x* axis) *versus* sharpened (*y* axis). Right, *MolProbity* scores for models using original first half-maps *versus* sharpened first half-maps.

Our tests using simulated data (§[Sec sec3.1]3.1) have indicated that map sharpening or blurring may be useful in refinement. To investigate this with the real experimental data we performed the following test. We selected models similarly to as described in §[Sec sec3.2.2]3.2.2, additionally requiring that independent half-maps had also been deposited by the researcher. This resulted in 76 entries. We performed test refinements against the first of the two half-maps and evaluated the refined model-to-data fit using the original second half-map that had not been used in any calculations. In two independent refinements, the first half-map was taken either as deposited or modified with *phenix.auto_sharpen* (Terwilliger, Sobolev *et al.*, 2018 ▸) to automatically optimally sharpen or blur the map. Fig. 7 ▸ shows the model–map correlation CC_mask_ for models refined against the original and sharpened first half-maps; the original second half-maps were used to compute the correlations. Overall, the CCs across all 76 cases are similar for refinement against the original first half-map and the sharpened first half-map. The refined models fit slightly but systematically better when using sharpened maps if the original model–map CC is low (<0.5) and systematically slightly worse if the original model–map correlation is higher (CC > 0.5). This agrees with the observation that target (7) allows the removal of large errors but may slightly distort exact models (§[Sec sec3.1.2]3.1.2). Also, we note that the *MolProbity* scores for models refined against sharpened maps are systematically better, but the difference is small.

#### 3.2.4. Re-refinement of the TRPV1 structure   

**Figure 8:**
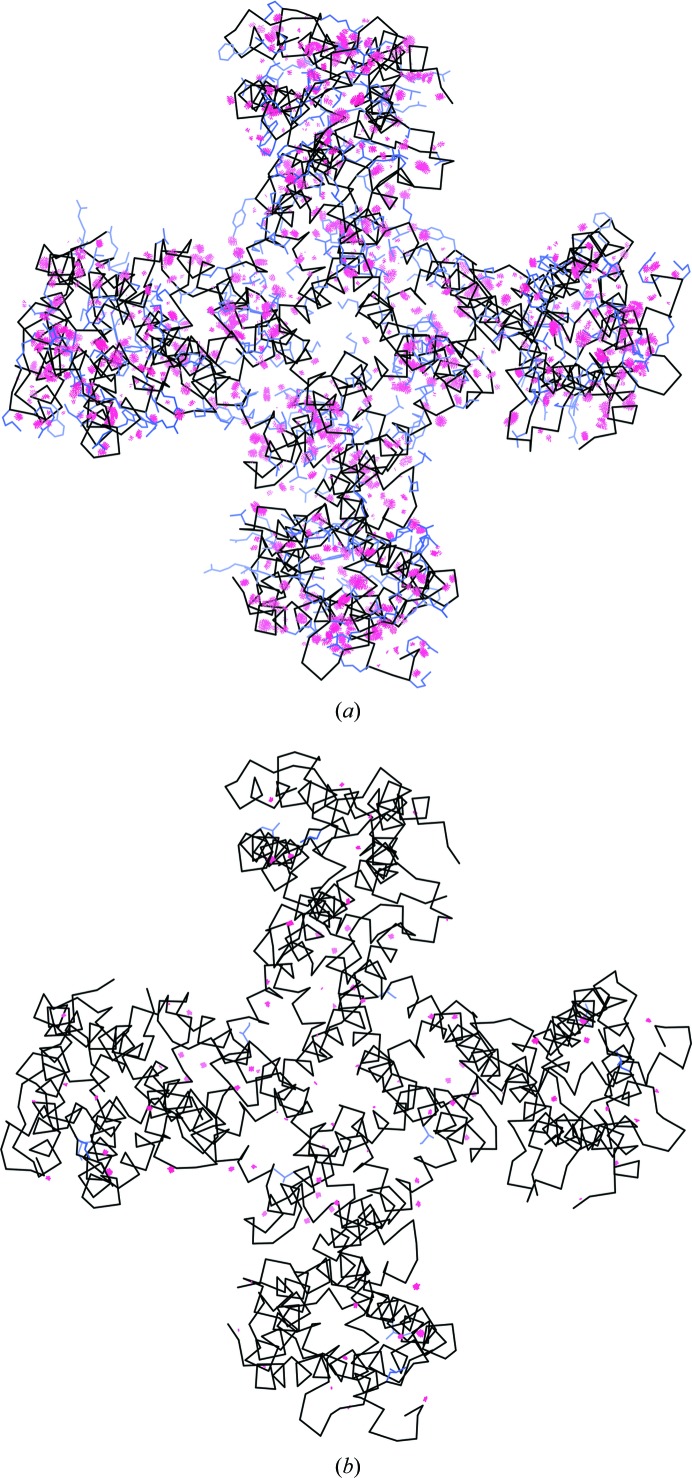
Backbone of the 3j5p model before (*a*) and after (*b*) refinement shown in black. The model before refinement contains a substantial number of steric clashes (indicated by red dots) and many side-chain rotamer outliers (blue side chains). Most clashes and rotamer outliers are resolved by *phenix.real_space_refine*. The images were created using the *KiNG* program (Chen *et al.*, 2009 ▸) from within *PHENIX*.

The structure of the TRPV1 ion channel (PDB entry 3j5p; EMDB code EMD-5778) was determined by single-particle cryo-EM (Liao *et al.*, 2013 ▸) at a resolution of 3.28 Å. The model was built manually and was not subjected to refinement. As the model was not refined it contains substantial geometry violations: the clashscore is high (∼100) and about one third of the side chains are identified as rotamer outliers (Table 1 ▸). More recently, the better resolved part of this structure has been re-evaluated using the same data (Barad *et al.*, 2015 ▸; PDB entry 3j9j; ankyrin domain not included). This involved some rebuilding and refinement using algorithms implemented in the *Rosetta* suite (DiMaio *et al.*, 2015 ▸). The resulting model has a much improved clashscore and *EMRinger* score (Barad *et al.*, 2015 ▸) and no rotamer outliers, yet the number of Ramachandran plot outliers has increased compared with the original model (Table 1 ▸). We performed a refinement of PDB entry 3j5p (the portion that matches PDB entry 3j9j) using *phenix.real_space_refine* with all default settings and automatically, with no manual intervention, using the original, deposited map. The refinement took about 3 min on a Macintosh laptop.^3^ Overall, the refined model is similar to PDB entry 3j9j (virtually no rotamer or Ramachandran plot outliers), the *EMRinger* score is improved further and the model-to-map correlation (CC_mask_) is increased compared with both PDB entries 3j5p and 3j9j. Notably, the *MolProbity* clashscore decreased from 100.8 to 5.6 as a result of the resolution of numerous steric clashes (Fig. 8 ▸).

**Figure 9:**
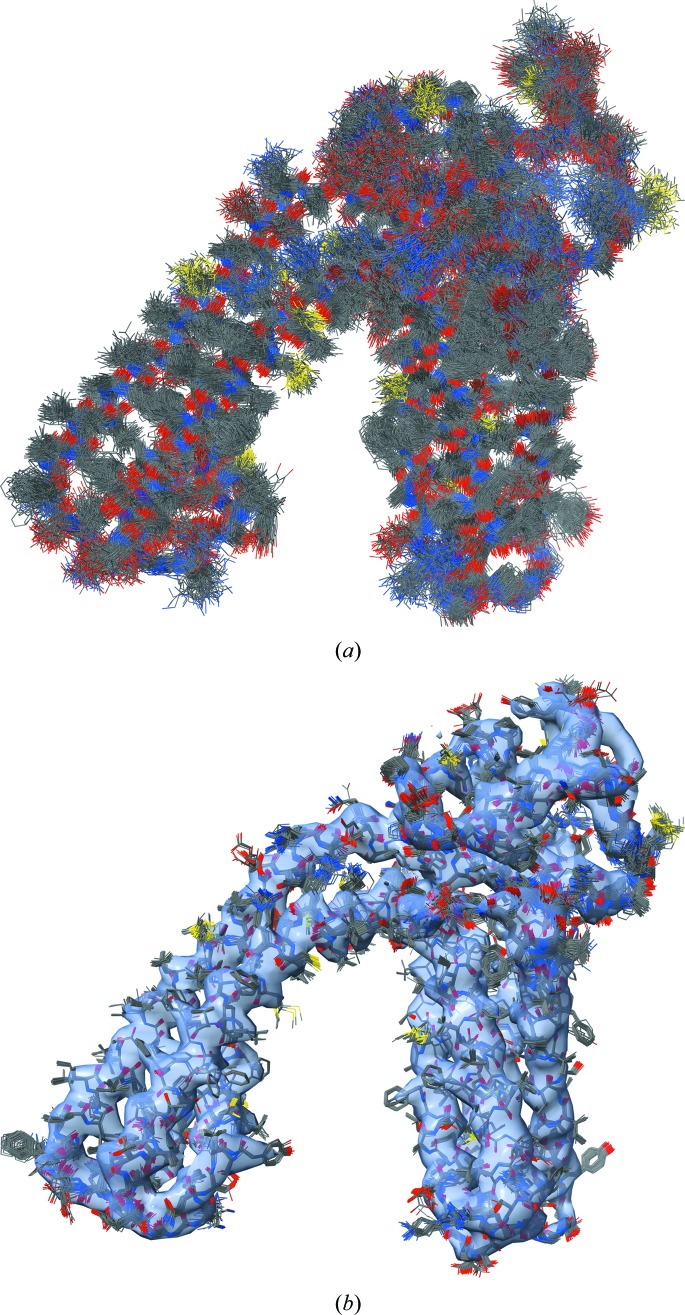
(*a*) Ensemble of perturbed 3j5p models; the r.m.s. deviation of each model from the initial model is 3 Å, showing chain *A* only. (*b*) Ensemble of refined models in the experimental map. The largest variation is observed in regions that lack density. The images were created using the *ChimeraX* program (Goddard *et al.*, 2018 ▸).

Modeling experimental data at resolutions below atomic (around 1–1.5 Å and better) may not be unambiguous (Terwilliger *et al.*, 2007 ▸). Therefore, it may be instructive to perform several trial refinements, each using the exact same settings but different (perturbed) input models. Here, we generated an ensemble of 100 perturbed models by running molecular-dynamics simulation (using *phenix.dynamics* tool) until the r.m.s. deviation between the starting and simulated models reached 3 Å (Fig. 9 ▸ *a*). We then refined all models using *phenix.real_space_refine* until convergence. This resulted in 100 refined models that are overall similar but vary locally (Fig. 9 ▸ *b*). This highlights the fact that a single-model representation of experimental data is an approximation and should not be taken too literally (for example, when it comes to measuring and reporting distances between atoms). Also, this test demonstrates the rather large convergence radius of *phenix.real_space_refine*: the average map–model correlation (CC_mask_) across all 100 refined models is 0.80, with the smallest and largest values being 0.79 and 0.81.

## 4. Conclusions   

Refinement of an atomic model against a map is increasingly important as the technique of cryo-EM rapidly develops. We have described the algorithms implemented in a new *PHENIX* tool, *phenix.real_space_refine*, that was specifically designed to perform such real-space refinements. RSR is a natural choice for cryo-EM, unlike crystallography, where real-space methods are complementary to Fourier-space refinement and are somewhat limited since crystallographic maps are almost always model-biased. Nevertheless, while this work was inspired by rapid advances in the field of cryo-EM and the increasing number of three-dimensional reconstructions that allow atomic models to be refined (as opposed to rigid-body docked), the implementation is not limited to cryo-EM and crystallographic maps can also be used.

The proposed real-space refinement procedure is fast owing to the use of an atom-centered refinement target function that has been shown to be efficient at all tested resolutions from 1 to 6 Å. Several options for key calculation steps, such as map interpolation, gradient calculation and preliminary processing of the target (experimental) map, are available with the default choices selected on the basis of extensive test calculations. The real-space refinement algorithm includes a fast and efficient search for the optimal relative weight of restraints, a procedure that is extremely challenging for reciprocal-space refinement. The refinement algorithm is robust, with no failures for any of the cryo-EM maps tested. For all test model refinements improvements are observed; in some cases these improvements are significant. Future developments of the algorithms will include methods to account for local variation in map resolution and a fast and accurate calculation of (5) for the final refinement cycles and efficient modeling of atomic displacements.

## Figures and Tables

**Table 1:** Summary of statistics for the original model (PDB entry 3j5p), that re-refined by Barad *et al.* (2015 ▸) (PDB entry 3j9j) and that re-refined by *phenix.real_space_refine* models

Metric	3j5p †	3j9j	3j5p † (*phenix.real_space_refine*)
CC_mask_	0.65	0.59	0.82
*EMRinger* score	1.2	2.6	3.3
R.m.s.d.			
Bonds (Å)	0.01	0.02	0.01
Angles (°)	1.50	1.10	1.44
Ramachandran plot (%)			
Favored	95.8	94.5	93.3
Allowed	4.2	3.3	6.7
Outliers	0	2.2	0
Rotamer outliers (%)	32.3	0	<1
Clashscore	100.8	2.7	5.6
C^β^ deviations	0	0	0

† No ankyrin domain.

## Footnotes

1 It is a widely known consequence of Parseval’s theorem [see, for example, Diamond (1971 ▸) or Arnold & Rossmann (1988 ▸)] that this is equivalent to a least-squares target between a full set of the corresponding complex Fourier coefficients; *CNS* (Brünger *et al.*, 1998 ▸) describes this as a ‘vector LS target’.

2 In crystallography, the set of the calculated Fourier coefficients usually coincides with that of the experimentally measured intensities.

3 For comparison of the CPU required by the two methods, we refer to Kim & Sanbonmatsu (2017 ▸).

## Appendix A Real-space targets and convolution

We show here that if the atoms all have the same shape, sampling a map at the positions of atomic centers, as in (7), can be made equivalent to the correlation function obtained by integrating or summing over the product of calculated and target densities, as in (3) or (5). Consider a simplified structure composed of a single atom. Looking for its best position according to (3) or (5) corresponds to seeking the position where the weighted average of the target map values (weighted by the atomic shape) inside a sphere centered at the trial atomic position is maximal. This calculation and check for the maximal value could be performed point by point. Alternatively, one can first calculate such averages for all grid points, replace the initial map values by these sums and then simply choose the maximum. From a mathematical point of view this averaging can be considered as a convolution and, if calculated simultaneously for the whole map, can be performed rapidly (Leslie, 1987 ▸; Urzhumtsev *et al.*, 1989 ▸). Checking the values of the averaged, *i.e.* blurred, map for their maximum corresponds to using targets (7) or (8). Below, we give a formal interpretation of these real-space targets.

Let *Z*_0_ *f*_0_(|**s**|; *B*_0_) be a scattering factor of some isotropic atom characterized by a *B*_0_ value and the electron number *Z*_0_. Let *Z*_0_ρ_0_(**r**; *B*_0_) be an image of this atom in the corresponding model map if it is placed at the origin. Both *Z*_0_ *f*_0_(|**s**|; *B*_0_) and *Z*_0_ρ_0_(**r**; *B*_0_) are spherically symmetric and related by Fourier transformation. If a hypothetical structure is composed of a single atom positioned at **r**_0_, the corresponding model map is

which can be seen as a convolution of a point scatterer at position **r**_0_ with the atomic shape. Owing to the spherical symmetry of ρ_0_(**r**; *B*_0_), the target function (3)

can be interpreted as a convolution of the target map with ρ_0_(**r**; *B*_0_) taken at point **r**_0_. Let {**F**_tar_(**s**)} be the set of Fourier coefficients corresponding to the target map ρ_tar_(**r**). By the convolution theorem, (10) is equal to the Fourier series of the corresponding Fourier coefficients,

Here, the map ρ_tar_0_(**r**; *B*_0_) is a Fourier series calculated with the coefficients **F**_tar_(**s**)*f*_0_(|**s**|; *B*_0_). In other words, instead of blurring the model map with the atomic shape and calculating the point-by-point product of the two maps, one may blur the experimental map and leave the model map unblurred, *i.e.* as a point map.

For a multi-atom model

At resolutions typical for bio-crystallography the shapes of macromolecular atoms are similar. If we additionally suppose that all of the atoms of the structure have the same (or similar) atomic displacement parameters *B_m_* = *B*_0_, then

using the function ρ_tar_0_(**r**; *B*_0_) calculated once in advance. This shows that in calculating (8) we in fact implicitly sharpen the target map using ρ_tar_(**r**) instead of ρ_tar_0_(**r**; *B*_0_). Even when using (8) as the target, it is likely to be beneficial to choose an optimal sharpening factor, just as the signal in map correlations can be improved.

If the difference in atomic *B* values cannot be neglected, one can calculate in advance a few maps ρ_tar_0_(**r**; *B*_*k*_) for a range of *B*-factor values *B_k_*, *k* = 1, …, *K*, and use the appropriate ρ_tar_0_(**r**_*m*_; *B*_*k*_) for a particular atom *m*,

If the atomic shapes are significantly different, as is the case for H atoms in neutron maps or negatively charged side chains in cryo-EM maps at high resolution, the approximation (13) can be used with *Z_m_* being a negative value, or the target map can be convoluted with the respective atomic shape (which can be negative) before the sum over the relevant atoms is calculated.

## Appendix B Three-dimensional interpolation used

### b1. General remarks

Using the atom-centered targets (7) and (8) requires an efficient and accurate interpolation of the maps calculated on three-dimensional regular grids. Not only the interpolated function values are needed but also the gradient. In this work, two options have been considered: trilinear (https://en.wikipedia.org/wiki/Trilinear_interpolation) and tricubic (https://en.wikipedia.org/wiki/Tricubic_interpolation). Both interpolation procedures, including the gradient calculation, are available through the *cctbx* software library (Grosse-Kunstleve *et al.*, 2002 ▸). Trilinear interpolation is the simplest and the easiest to understand. Its major disadvantage is that, by construction, the minimum of the interpolated function is always at one of the corners of the box of interpolation. Since the map grid step is usually larger that the accuracy of atomic positions required, this can impact the optimization procedure and results. For this reason, the tricubic interpolation has been chosen as the default method. Other interpolations have also been tried but are not discussed in this work. In the following, we first describe the interpolation procedures inside the unit cube and then adapt the results and the procedures to an arbitrary regular tridimensional grid.

### b2. Tricubic interpolation inside a unit cube

Let us consider an interpolation inside a unit cube, 0 ≤ *x* < 1, 0 ≤ *y* < 1, 0 ≤ *z* < 1. We search for a function in the form

This function is cubic with respect to any of its three variables, giving expressions for the partial derivatives

One can calculate all 64 coefficients in advance and use them for further calculations (Lekien & Marsden, 2005 ▸). Alternatively, one can build an interpolation for the coordinate *x*, then for the coordinate *y* and finally for the coordinate *z* (in any order of variables). To build interpolation (16) eight values from the cube corners are insufficient and either values from the neighboring grid points (the corners of the neighboring cubes) or derivatives in the corners of the unit cube are required. In the following, *f_pqr_* with integers *p*, *q*, *r* stand for the grid function values *f*(*p*,* q*, *r*).

Firstly, we define a cubic interpolation

of a function *f*(*x*) of one variable in the interval (0, 1) for which its values are known in the integer grid nodes, *f*_−1_ = *f*(−1), *f*_0_ = *f*(0), *f*_1_ = *f*(1), *f*_2_ = *f*(2). We notate this interpolation by int3(*x*; *f*_−1_, *f*_0_, *f*_1_, *f*_2_) and its derivative by gint3(*x*; *f*_−1_, *f*_0_, *f*_1_, *f*_2_), as they are called in *cctbx*:

The coefficients of this approximation are derived below. The procedure of the tricubic interpolation then becomes a suite of operations:

where *p* and *q* are integers −1, 0, 1 or 2, then

and finally

The last three expressions are redundant and only one of them can be calculated. However, the expressions previous to them are necessary to calculate partial derivatives as

The coefficients of the one-dimensional cubic interpolation (17) can be chosen using various considerations. The possibility taken as the default choice in the current software version is to build a cubic function  such that it and its first derivative coincide with *f*(*x*) and with *f*′(*x*), respectively, at points 0 and 1. Since the *f*′(0) and *f*′(1) values are unknown, they are estimated as

This gives the coefficients of (17) in the form

### b3. Tricubic interpolation on a regular grid

Now let a function *f*(*x*, *y*, *z*) be defined in fractional coordinates on a grid with the step *d_x_* = *N_x_* ^−1^, *d_y_* = *N_y_* ^−1^, *d_z_* = *N_z_* ^−1^. Let us consider a point (*x_g_*, *y_g_*, *z_g_*) and a box of this grid that this point belongs to,

with *n_x_*, *n_y_*, *n_z_* being integer numbers. We introduce intermediate variables rescaling this ‘box’ to a unit cube as

and apply the procedure (19)–(21) described above. According to (26), the respective derivatives are
